# Factors influencing the psychological help-seeking behaviour of migrant older adults in China: a cross-sectional study

**DOI:** 10.3389/fpubh.2025.1708061

**Published:** 2026-01-09

**Authors:** Fang Li, Yating Wang, Na Zhou, Tianle Xiang, Jianlin Lou, Libing Zhang, Meijuan Cao

**Affiliations:** 1 School of Nursing, Huzhou University, Huzhou, Zhejiang, China; 2 Huzhou Key Laboratory of Precise Prevention and Control of Maior Chronic Diseases, Huzhou University, Huzhou, China

**Keywords:** help-seeking behaviour, mental health, migrant older adults, COM-B model, influencing factors

## Abstract

**Background:**

Psychological help-seeking behaviour is a key determinant of mental health outcomes. However, studies on psychological help-seeking behaviour that focus on older adults are very limited, especially for migrant older adults. Identifying contributing factors could inform tailored interventions to enhance help-seeking behaviour amongst this vulnerable population. This study aimed to explore the current status and influencing factors of psychological help-seeking behaviour amongst migrant older adults under the guidance of the COM-B model.

**Methods:**

A cross-sectional study was conducted with a convenience sample of 345 migrant older adults from Huzhou, Zhejiang Province, China, and the participants were assessed using the Depression Anxiety Stress Scale (DASS-21), General Help-Seeking Questionnaire (GHSQ), Actual Help-Seeking Questionnaire (AHSQ), Attitudes Towards Seeking Professional Psychological Help Scale–Short Form (ATSPPH-SF), Questionnaire of Stigma for Seeking Professional Psychological Help (SSPPH), Mental Health Literacy Scale (MHLS), and Perceived Social Support Scale (PSSS).

**Results:**

The analysis found that 146 participants (42.32%) reported psychological distress, of whom 99 (67.81%) sought help, whereas only 11 (11.11%) used professional services, highlighting the markedly low utilisation of professional psychological support. Multiple linear regression revealed that sex, reemployment/volunteering, psychological distress, mental health literacy, perceived social support and the intention to seek nonprofessional help were significantly associated with nonprofessional help seeking. Psychological distress, mental health literacy and the intention to seek professional help were associated with professional help seeking.

**Conclusion:**

A substantial proportion of migrant older adults experiencing psychological distress did not seek professional help, revealing gaps in help-seeking behaviour. This study highlights that targeted interventions should be developed to reduce structural barriers and enhance the help-seeking motivation, opportunity and capacity of older migrant adults.

## Introduction

With the accelerating urbanisation and population ageing, increasing numbers of older adults migrate from one city to another within their countries for work, family reunification, or childcare support (1). In China, the population of migrant older adults reached 49.3 million in 2020 and is experiencing rapid growth (2). Migrant older adults face dual vulnerabilities related to ageing and migration, such as physiological decline and limited social adaptability. Compared with their nonmigrant counterparts, migrant older adults have a significantly greater risk of mental health problems (3). The prevalence of depression in this group is estimated at 20–30%, and their suicide rate is three times higher (4). These disparities are linked to disrupted living environments, language barriers, cultural differences, and weakened social networks, leading to emotional distress, such as loneliness, depression, and anxiety (2, 3). Additionally, caregiving responsibilities and household support obligations may compound their psychological burden (5). Therefore, against the backdrop of increasingly prominent mental health issues, the psychological help-seeking behaviour of migrant older adults has become a critical topic that warrants in-depth investigation.

Psychological help-seeking behaviour refers to individuals’ efforts to seek understanding, support, or treatment when experiencing emotional or mental health difficulties. This includes both professional (e.g., mental health professionals) and nonprofessional sources (e.g., family members or friends) (6). Research has demonstrated the significance of psychological help-seeking behaviour in preventing the progression of various mental illnesses and suicidal behaviour (6). According to the Theory of Planned Behaviour, help-seeking intention is shaped by an individual’s attitudes, subjective norms, and perceived behavioural control (7), whilst the Health Belief Model highlights perceived need, benefits, and barriers as key determinants of such behaviour (8). Furthermore, drawing on Goffman’s theory of social stigma, individuals who internalize negative societal labels regarding mental illness may experience shame or fear of social judgement, which significantly discourages them from seeking psychological assistance (9). Recent studies have also confirmed that social stigma is closely linked to lower psychological well-being and reduced help-seeking tendencies (10). In China, only about 10–15% of older adults with depressive symptoms seek professional psychological assistance (11, 12), and this proportion is even lower amongst migrant older adults due to cultural beliefs, stigma, and limited knowledge of available services (13, 14). In addition, unequal distribution and limited accessibility of mental health services between urban and rural areas further hinder help-seeking (15). Consequently, inadequate help-seeking not only exacerbates psychological distress but also increases the social and healthcare burden (16, 17).

However, research on psychological help-seeking behaviour amongst older adults remains limited, particularly for migrant older adults (17, 18). Most existing studies on migrant older adults have focused on social integration and access to older adult services (19), whilst studies on psychological help-seeking have mainly concentrated on populations such as adolescents, perinatal women (20), and university students (21). Psychological help seeking is a complex behavioural process influenced by multiple factors. Some studies focus on demographic, psychological, and sociocultural factors affecting help-seeking behaviour. For example, gender, educational level, and age have all been shown to affect psychological help-seeking behaviour (22). Women tend to seek help more than men do (23), and individuals with higher educational attainment are more likely to recognise mental health issues and actively seek support (24). The effect of age, by contrast, remains controversial—whilst Sorkin et al. suggest that older age hinders psychological help seeking, other evidence indicates that older adults may be more capable of overcoming barriers to seeking help (25).

In terms of psychological factors, the severity of psychological distress, level of mental health literacy, attitudes towards psychological help seeking, and the intention to seek help all play critical roles (16, 26, 27). Improved mental health literacy has been linked to a greater willingness to seek help, whereas increased distress may also prompt individuals to reach out. Nonetheless, even when individuals express positive attitudes towards psychological help seeking, actual behaviour may still be hindered by stigma, fear, or embarrassment. Sociocultural factors, such as cultural norms, the availability of social support, and stigma towards mental illness, also significantly impact psychological help-seeking behaviour (21, 27–29). Perceptions of mental health and the willingness to seek help vary across cultural contexts. The level of social support plays a crucial role in facilitating access to assistance, whereas perceived stigma may discourage individuals from seeking help due to fear of discrimination (29).

The Capability, Opportunity, Motivation–Behaviour (COM-B) model, developed by Michie et al. as part of the Behaviour Change Wheel framework, provides a comprehensive theoretical basis for understanding behavioural change (30). The model posits that any behaviour results from the dynamic interaction amongst three components: capability (the individual’s psychological and physical ability to perform the behaviour), opportunity (the external factors that enable or prompt the behaviour), and motivation (the internal processes that direct and energise the behaviour) (30). By encompassing reflective and automatic motivation, psychological and physical capability, and social and physical opportunities, the COM-B model allows for a flexible and multilevel analysis of both facilitating and inhibiting factors, as well as the mechanisms underlying behavioural performance. In addition, contemporary interdisciplinary research has applied behavioural models from psychology, public health, and sociology to mental health, emphasising how individual cognition, social norms, and environmental constraints jointly shape help-seeking behaviour. Integrating these perspectives complements COM-B by highlighting both personal and structural determinants of mental health actions (31, 32). The COM-B model has been applied to explore psychological help-seeking in different populations. Ma et al. examined barriers and facilitators amongst individuals with schizophrenia using a qualitative COM-B approach (33), whilst Nguyen et al. investigated psychiatrists’ access to mental health services, highlighting key personal and environmental influences on help-seeking behaviour (34). Overall, few studies have systematically examined the factors influencing psychological help-seeking amongst migrant older adults using a comprehensive theory-driven framework.

Accordingly, this study aims to holistically examine psychological help-seeking amongst migrant older adults and to explore the influence of capability (e.g., mental health literacy, psychological distress), opportunity (e.g., social support, stigma), and motivation (e.g., attitudes, help-seeking intentions) within the COM-B framework. The findings are intended to offer both theoretical insights and practical guidance for improving psychological help-seeking behaviour, promoting mental health service utilisation, and enhancing psychological well-being amongst older migrant adults in the context of global trends in ageing and population migration.

## Materials and methods

### Study design and participants

This study used a cross-sectional design. Considering the research objectives and the feasibility of sample recruitment, convenience sampling was employed. Between April and August 2024, a total of 345 migrant older adults were recruited from community service centres, public parks, and open spaces in Huzhou, Zhejiang Province. According to the Seventh National Population Census of China (35), the total population of Huzhou City reached approximately 3.37 million, of which 27.8% were migrants from other regions within China. As a typical net-inflow city, Huzhou attracts older adults from both within and outside the province due to its favourable socioeconomic conditions, accessible healthcare, comprehensive public services, and a relatively pleasant living environment, reflecting its strong appeal to the migrant population (17, 33).

Under China’s Household Registration System (Hukou System), formally known as the People’s Republic of China Household Registration Administration System, individuals are assigned a permanent household registration location at birth (2, 36). In this study, migrant older adults were defined as individuals aged ≥60 years who had resided in the study area for at least 6 months without transferring their hukou.

The inclusion criteria were as follows: (1) age ≥60 years; (2) a duration of migration >6 months without hukou transfer; and (3) voluntary participation with written consent. The exclusion criteria were as follows: (1) severe cognitive impairment; (2) serious physical illness; and (3) major language or communication disorders.

According to Kendall’s sampling estimation method (37), the sample size should be 5–10 times the number of variables. With 32 variables, the estimated range in this study was 160–320. Accounting for a 10% attrition rate, the final target sample size was 176–352.

In this study, variables other than general sociodemographic characteristics were systematically organised according to the COM-B model to provide a coherent theoretical foundation for the analysis. Capability included psychological distress and mental health literacy, reflecting individuals’ ability to recognise and manage mental health needs. Opportunity encompassed social support and stigma towards mental illness, representing environmental and social facilitators or barriers. Motivation comprised attitudes towards psychological help seeking and help-seeking intentions, capturing internal drivers that promote or inhibit such behaviour.

### Measures

#### Sociodemographic characteristics

A self-designed questionnaire developed according to the study objectives and literature review collected demographic information, including gender, age, education level, marital status, average monthly household income, migration characteristics, self-rated health, and recent emotional status. Self-rated health and emotional status were assessed via a 3-point Likert scale.

#### Psychological distress

It was measured using the 21-item Depression Anxiety and Stress Scale (DASS-21), originally developed by Lovibond (57) and adapted for Chinese populations by Gong et al. (38). The scale contains 21 items rated on a 4-point Likert scale (0 = “Did not apply to me at all” to 3 = “Applied to me very much or most of the time”). The Cronbach’s alpha for the scale was 0.890. Scores for each dimension are doubled to yield total scores ranging from 0 to 126, with higher scores indicating greater distress. Screening criteria for psychological distress were defined as follows: participants scoring 10–13, 14–20, and ≥21 on the depression subscale were classified as having mild, moderate, and severe depression, respectively; those scoring 8–9, 10–14, and ≥15 on the anxiety subscale were classified as having mild, moderate, and severe anxiety, respectively; and those scoring 15–18, 19–25, and ≥26 on the stress subscale were classified as having mild, moderate, and severe stress, respectively. Participants who met the threshold for any of these dimensions were considered to have psychological distress and were included in subsequent analyses (38). The scale has been widely used amongst older adult populations, and previous studies have confirmed its good validity and reliability in this demographic (39). In this study, the Cronbach’s alpha was 0.849.

#### Psychological help-seeking intentions

The General Help-Seeking Questionnaire (GHSQ), developed by Wilson et al. was used to assess individuals’ intentions to seek help from various sources for emotional problems (39). The scale comprises 11 items rated on a 7-point Likert scale (1 = “Extremely unlikely” to 7 = “Extremely likely”), with the “no help-seeking” item reverse-coded. The Cronbach’s alpha for the scale was 0.810. Total scores range from 11 to 77, with higher scores indicating stronger help-seeking intentions. Intentions were classified as professional (e.g., mental health professionals, general practitioners), nonprofessional (e.g., family, friends, neighbours), or not seeking help. The scale has been successfully used in older adult populations, with established studies confirming its good validity and reliability in this demographic (40). In this study, Cronbach’s alpha was 0.773.

#### Psychological help-seeking behaviour

It was assessed using a self-developed Actual Help-Seeking Questionnaire (AHSQ), adapted from the GHSQ to measure behavioural responses to psychological distress (39). A pilot study was conducted with 62 migrant older adults to refine and revise the questionnaire, ensuring that the help sources matched the help-seeking intentions identified in this group. The Cronbach’s *α* coefficient for the pilot study was 0.782. The scale contains 11 items rated on a 7-point Likert scale (1 = “Extremely unlikely” to 7 = “Extremely likely”) to assess the frequency and preference for different help sources. Scores range from 11 to 77 and encompass three dimensions: nonprofessional, professional, and no help seeking. In this study, Cronbach’s alpha was 0.750.

#### Attitudes towards professional psychological help

It was measured using the Attitudes Towards Seeking Professional Psychological Help Scale–Short Form (ATSPPH-SF), developed by Fisher and translated into Chinese by Fang et al. (41). This 10-item scale covers two dimensions: acceptability and value/need. The Cronbach’s alpha for the scale was 0.800. The items are rated on a 4-point Likert scale (0 = “Disagree” to 3 = “Agree”), with total scores ranging from 0 to 30. Higher scores indicate more positive attitudes. It has demonstrated good internal consistency and construct validity amongst Chinese older adults (41). In this study, Cronbach’s alpha was 0.805.

#### Stigma for seeking professional psychological help

It was measured using the Questionnaire of Stigma for Seeking Professional Psychological Help (SSPPH), developed by Chinese scholars Hao et al. (42). The SSPPH includes 10 items across self-stigma and public stigma subscales, rated on a 5-point Likert scale (1 = “Strongly disagree” to 5 = “Strongly agree”). The Cronbach’s alpha for the scale was 0.832. Total scores range from 10 to 50, with higher scores indicating greater perceived stigma. In this study, Cronbach’s alpha was 0.854.

#### Multicomponent mental health literacy

It was assessed using the multicomponent mental health literacy measure (MMHL), developed by Jung and adapted for Chinese populations by Ming et al. (43). The scale consists of 22 items assessing knowledge, beliefs, and awareness of mental health resources. The Cronbach’s alpha for the scale was 0.810. The knowledge and belief items use dichotomous scoring (correct = 1; incorrect/uncertain = 0), whereas the resource items are scored as yes = 1 and no = 0. Total scores range from 0 to 26, with higher scores indicating better mental health literacy. It has shown acceptable psychometric properties in Chinese adult samples (43). In the present study, Cronbach’s alpha was 0.804.

#### Perceived social support

Originally developed by Zimet and translated by Huang et al. the Perceived Social Support Scale (PSSS) assesses perceived support from family and nonfamily sources (44). The scale contains 12 items rated on a 7-point Likert scale (1 = “Strongly disagree” to 7 = “Strongly agree”), with total scores ranging from 12 to 84. The Cronbach’s alpha for the scale was 0.921. Higher scores indicate greater perceived social support. Scores ≥61 (total), ≥20 (family), and ≥40 (nonfamily) reflect high support. It has demonstrated excellent internal consistency and construct validity in Chinese samples (44). In this study, Cronbach’s alpha was 0.905.

### Data collection

A paper-based questionnaire survey was administered to migrant older adults by trained postgraduate nursing students. Before the formal survey, all investigators completed 1 week of standardised training covering the survey content, administration procedures, precautions, and communication skills tailored for older adults. Data were collected through onsite visits to community health service centres, community management offices, community parks, squares, and other public areas in the main urban districts and economic development zones of Huzhou. A total of 370 questionnaires were distributed, 357 were returned, and 345 were deemed valid, yielding an effective response rate of 93.2% and a refusal rate of 6.8%. During the data collection process, researchers provided face-to-face explanations regarding the purpose, significance, and instructions for completing the questionnaire. Participants were informed that participation was voluntary and anonymous. After obtaining informed consent, participants were encouraged to complete the questionnaire independently. For participants with limited literacy or reading difficulties, the researchers read each item aloud, recorded verbal responses, and verified the answers upon completion. Both psychological distress and psychological help-seeking intention were assessed with reference to the past 4 weeks to ensure temporal consistency. All questionnaires were collected immediately onsite and verified for completeness. The completed questionnaires were securely stored in a locked cabinet, with access restricted to authorised members of the research team to ensure data confidentiality.

### Statistical analysis

All valid questionnaire data were double-entered and checked independently by two researchers using Epidata 3.1. Data analyses were performed via SPSS version 26.0 and AMOS version 28.0. A two-sided alpha level of 0.05 was used to determine statistical significance (*p* < 0.05). Variables with statistical significance in the univariate analysis were subsequently entered into multiple linear regression models to identify factors associated with psychological help seeking. Multiple linear regression was chosen because it allows simultaneous examination of the effects of multiple independent variables on continuous outcome measures, and it provides interpretable estimates of associations whilst controlling for potential confounders. Despite the skewness of professional help-seeking scores (median = 0), the model provides useful insight into the relative contribution of each predictor. To ensure robustness, we inspected the correlation matrix of independent variables and found that no pairwise correlations exceeded 0.7. Tolerance values were all above 0.45, further supporting the absence of problematic multicollinearity. The maximum VIF was 1.470, indicating that independent variables were sufficiently distinct to provide reliable regression estimates.

## Results

### Descriptive statistics

A total of 370 questionnaires were distributed, and 345 valid responses were obtained (93.2%). The participants’ ages ranged from 55 to 92 years, with a mean age of 66.29 ± 7.72 years. The majority of participants were female (57.7%). Most had primary school education or below (60.6%), were married (86.7%), and held rural household registration (80.3%). The main reason for migration was caregiving for grandchildren (40.6%). In terms of residence, most had lived in the current location for 1–3 years (26.1%). The most common monthly income range was $100–299 (33.0%). Most reported no religious affiliation (82.9%), lived with other family members (52.7%), and received a moderate level of care from their children (59.4%). Over half (65.8%) did not engage in reemployment or volunteer activities. Regarding emotional status, the majority rated it as average (53.6%). Detailed demographic and health characteristics are presented in Table 1.

**Table 1:** Univariate analysis of nonprofessional and professional psychological help-seeking behaviours across different characteristics amongst migrant older adults (*n* = 345).

Variables	*N*	%	Nonprofessional psychological help-seeking behaviour			Professional psychological help-seeking behaviour			
			Total score ( $\bar{x}$ ± s)	*t*/*F*	*p*	Total score		*Z*/*χ*^2^	*p*
						( $\bar{x}$ ± s)	*M* (QR)		
Age (year)									
55–64	166	48.1	2.46 ± 2.32	5.356	0.013*	0.09 ± 0.41	0 (0)	5.356^b^	0.069
65–74	124	35.9	1.93 ± 1.98			0.01 ± 0.09	0 (0)		
≥75	55	16.0	1.58 ± 1.90			0.02 ± 0.16	0 (0)		
Gender									
Male	146	42.3	1.27 ± 1.71	−6.943	<0.001**	0.03 ± 0.20	0 (0)	−1.034^a^	0.301
Female	199	57.7	2.75 ± 2.25			0.07 ± 0.35	0 (0)		
Educational level									
Primary and below	209	60.6	1.97 ± 1.83	0.941	0.448	0.02 ± 0.17	0 (0)	1.061^b^	0.398
Junior middle	101	29.3	2.46 ± 2.64			0.10 ± 0.44	0 (0)		
Senior high	31	9.0	2.23 ± 2.39			0.03 ± 0.18	0 (0)		
College and above	4	1.1	1.50 ± 3.00			0.50 ± 1.00	0 (1.5)		
Marital status									
Married	299	86.7	2.27 ± 2.21	10.195	0.002^**^	0.04 ± 0.29	0 (0)	4.287^b^	0.117
Unmarried/Divorced	6	1.7	2.00 ± 2.45			0.33 ± 0.82	0 (0.5)		
Widowed	40	11.6	1.10 ± 1.37			0.05 ± 0.22	0 (0)		
Household registration									
Rural	277	80.3	2.13 ± 2.13	0.042	0.967	0.04 ± 0.29	0 (0)	−0.108^a^	0.993
Urban	68	19.7	2.12 ± 2.32			0.07 ± 0.32	0 (0)		
Monthly income ($)									
<100	84	24.3	2.06 ± 2.09	1.158	0.328	0.08 ± 0.42	0 (0)	3.383^b^	0.336
100–299	114	33.0	1.88 ± 1.91			0.01 ± 0.09	0 (0)		
300–499	109	31.6	2.29 ± 2.30			0.06 ± 0.31	0 (0)		
>500	38	11.0	2.55 ± 2.59			0.05 ± 0.32	0 (0)		
Religious affiliation									
None	286	82.9	1.95 ± 1.97	7.560	0.004^**^	0.04 ± 0.24	0 (0)	0.785^b^	0.376
Buddhism	50	14.5	2.52 ± 2.49			0.08 ± 0.44	0 (0)		
Christianity	9	2.6	5.67 ± 3.00			0.22 ± 0.67	0 (0)		
Living arrangements									
Living alone	10	2.9	1.40 ± 2.07	1.434	0.233	0.30 ± 0.68	0 (0.25)	19.025^b^	<0.001^**^
Living with spouse	150	43.5	2.11 ± 2.15			0.05 ± 0.27	0 (0)		
Living with family (including children)	182	52.7	2.15 ± 2.18			0.03 ± 0.28	0 (0)		
Others	3	0.9	4.33 ± 1.53			0.33 ± 0.58	0 (0)		
Children’s concern									
Always/often	130	37.7	2.42 ± 2.40	2.189	0.114	0.05 ± 0.31	0 (0)	1.486^b^	0.476
Sometimes	205	59.4	1.97 ± 2.00			0.04 ± 0.29	0 (0)		
Rarely/None	10	2.9	1.50 ± 1.90			0.10 ± 0.32	0 (0)		
Previous experience of seeking professional psychological help									
No	309	89.6	1.99 ± 2.02	12.484	<0.001^**^	0.01 ± 0.10	0 (0)	150.418^b^	<0.001^**^
Self	10	2.9	5.40 ± 2.12			1.20 ± 1.03	1 (2)		
Friend(s)	26	7.5	2.54 ± 2.80			0.08 ± 0.39	0 (0)		
Reemployment/volunteering									
No	118	65.8	1.88 ± 1.92	2.737	0.007^**^	0.04 ± 0.28	0 (0)	−0.172^a^	0.863
Yes	227	34.2	2.60 ± 2.50			0.06 ± 0.33	0 (0)		
Length of residence in current location									
6 months to 1 year	44	12.8	2.39 ± 2.72	2.978	0.021	0.02 ± 0.15	0 (0)	3.195^b^	0.526
1–3 years	90	26.1	2.30 ± 2.00			0.06 ± 0.35	0 (0)		
3–5 years	85	24.6	2.36 ± 2.10			0.09 ± 0.40	0 (0)		
5–10 years	65	18.9	2.05 ± 2.36			0.03 ± 0.25	0 (0)		
>10 years	61	17.7	1.44 ± 1.670			0.02 ± 0.13	0 (0)		
Migration range									
Across provinces	145	42.0	1.83 ± 1.85	2.497	0.086	0.02 ± 0.25	0 (0)	10.736^b^	0.005^**^
Across cities	49	14.2	2.41 ± 2.44			0.16 ± 0.51	0 (0)		
Across districts	151	43.8	2.32 ± 2.32			0.04 ± 0.23	0 (0)		
Migration reason									
Caring for grandchildren	140	40.6	2.53 ± 2.28	4.216	0.007^**^	0.03 ± 0.27	0 (0)	2.513^b^	0.473
Work/farming	80	23.2	1.96 ± 2.18			0.09 ± 0.40	0 (0)		
Retirement	99	28.7	1.61 ± 1.88			0.05 ± 0.26	0 (0)		
Seeking medical care	26	7.5	2.46 ± 2.08			0.04 ± 0.20	0 (0)		
Self-rated emotional status									
Good	136	39.4	2.23 ± 2.25	1.723	0.180	0.01 ± 0.09	0 (0)	9.768^b^	0.008^**^
Average	185	53.6	1.99 ± 2.08			0.05 ± 0.29	0 (0)		
Poor	24	7.0	2.83 ± 2.43			0.25 ± 0.74	0 (0)		

^a^Mann–Whitney U test; ^b^Kruskal–Wallis H test; *p < 0.05; **p < 0.01.

### Psychological help-seeking behaviour status and univariate analysis

Amongst the 345 migrant older adults surveyed, 146 (42.3%) exhibited varying degrees of psychological distress; however, only 22 participants (15.1%) were aware that they were experiencing emotional problems. Amongst those individuals with psychological distress, 99 (67.8%) had engaged in some form of psychological help-seeking behaviour for emotional or psychological issues in the past 4 weeks, yet only 11 (7.5%) had sought help from professional mental health services. The primary sources of psychological help were spouses (56.8%), followed by children (47.8%) and friends (23.5%).

Univariate analyses, including independent sample t tests and one-way ANOVA, revealed that nonprofessional psychological help-seeking behaviour amongst migrant older adults differed significantly by gender, age, marital status, religious belief, prior experience with professional psychological help, engagement in reemployment or volunteer activities, duration of stay in the host city, and reasons for migration (all *p* < 0.05). Nonparametric tests revealed that professional psychological help-seeking behaviours differed significantly across variables, such as living arrangement, prior experience with professional psychological help, migration scope, and recent emotional self-evaluation (*p* < 0.05) (Table 1).

### Multiple regression analysis

Multiple linear regression analyses were conducted with professional and nonprofessional psychological help-seeking behaviours as dependent variables and variables showing significant associations in univariate analyses as independent variables.

At the demographic level, sex and engagement in reemployment or volunteer activities were significantly associated with nonprofessional psychological help-seeking behaviour. Specifically, one-unit increase in sex (female = 1) was significantly associated with an increase in nonprofessional psychological help seeking (*β* = 0.827, *p* < 0.001). One-unit increase in engagement in reemployment or volunteer activities was also significantly associated with an increase in nonprofessional help seeking (*β* = 0.366, *p* = 0.031).

At the capability level, psychological distress and mental health literacy were significant predictors of both professional and nonprofessional psychological help-seeking behaviours. One-unit increase in psychological distress was significantly associated with an increase in nonprofessional help seeking (*β* = 0.046, *p* < 0.001) and professional help seeking (*β* = 0.007, *p* < 0.001). Likewise, one-unit increase in mental health literacy was significantly associated with an increase in nonprofessional help seeking (*β* = 0.151, *p* < 0.001) and professional help seeking (*β* = 0.022, *p* < 0.001).

At the opportunity level, perceived social support was a significant predictor of nonprofessional help seeking. One-unit increase in perceived social support was significantly associated with an increase in nonprofessional psychological help seeking (*β* = 0.040, *p* = 0.003).

At the motivation level, intention strongly influenced help-seeking behaviours. One-unit increase in the intention to seek nonprofessional psychological help was significantly associated with an increase in nonprofessional help seeking (*β* = 0.258, *p* < 0.001), making it the strongest predictor at this level. Similarly, one-unit increase in the intention to seek professional psychological help was significantly associated with an increase in professional help-seeking behaviour (*β* = 0.028, *p* < 0.001) (Tables 2, 3).

**Table 2:** Multiple linear regression analysis of factors associated with nonprofessional psychological help-seeking behaviour amongst migrant older adults (*n* = 345).

Variables	*B*	SE	*β*	*t*	*p*	95.0% CI	
(Constant)	−9.068	0.843		−10.760	<0.001	−10.726	−7.410
Sex (ref: male)	0.827	0.168	0.189	4.929	<0.001	0.497	1.156
Reemployment/volunteering (ref: No)	0.366	0.169	0.080	2.162	0.031	0.033	0.700
Psychological distress	0.046	0.007	0.268	6.540	<0.001	0.032	0.060
Mental health literacy	0.151	0.024	0.260	6.254	<0.001	0.103	0.198
Social support	0.040	0.014	0.129	2.944	0.003	0.013	0.067
Nonprofessional psychological help-seeking intentions	0.258	0.022	0.479	11.657	<0.001	0.215	0.302

*R* = 0.746, *R*^2^ = 0.557, *F* = 70.881, *p* < 0.001.

**Table 3:** Multiple linear regression analysis of factors associated with professional psychological help-seeking behaviour amongst migrant older adults (*n* = 345).

Variables	*B*	SE	*β*	*t*	*p*	95.0% CI	
(Constant)	−0.363	0.043		−8.547	<0.001	−0.447	−0.280
Psychological distress	0.007	0.001	0.307	6.643	<0.001	0.005	0.009
Mental health literacy	0.022	0.004	0.279	5.463	<0.001	0.014	0.030
Professional psychological help-seeking intentions	0.028	0.007	0.209	4.097	<0.001	0.014	0.041

*R* = 0.526, *R*^2^ = 0.277, *F* = 43.448, *p* < 0.001.

## Discussion

This study revealed that a substantial proportion of migrant older adults in Huzhou, China, experienced psychological distress, yet their psychological help-seeking rate—particularly for professional services—remained low. Amongst the migrant older adults surveyed, the proportion experiencing psychological distress was substantial (42.3%), yet awareness of their own emotional or mental health issues remained very low (15.1%). This gap between experienced distress and problem recognition highlights a generally low level of mental health literacy in this population. Amongst the 146 migrant older adults experiencing varying degrees of psychological distress, 99 individuals (67.8%) engaged in nonprofessional psychological help-seeking behaviours in the past 4 weeks, slightly lower than the 68.7% reported amongst depressed older Chinese Americans Kong et al. (45). In contrast, only 11 participants (7.5%) sought professional psychological support, markedly lower than the 37.5% professional help-seeking rate amongst depressed older Chinese Americans Kong et al. (45), slightly lower than the 10.6% reported amongst older adults with suicidal ideation in China (46), and comparable to the 7.65% observed amongst depressed older adults in Mexico (47). Migrant older adults thus appear to face considerable challenges in recognising mental health problems and accessing professional services. They tend to rely on nonprofessional sources, such as spouses, children, or friends, rather than general practitioners or mental health professionals. This preference may stem from limited access to health care services, underdeveloped mental health systems, and persistent stigma surrounding mental illness.

From a theoretical perspective, the psychological help-seeking behaviours of migrant older adults can be understood through the COM-B health behaviour model and social network theory. The COM-B model posits that help-seeking is shaped by individuals’ capability, opportunity, and motivation: limited mental health literacy reduces capability, stigma constrains opportunity, and negative attitudes reduce motivation, collectively limiting professional help-seeking. Social network theory further explains the reliance on informal sources, as existing family and community ties provide accessible but nonprofessional support, influencing both help-seeking choices and perceived needs (48). In line with these frameworks, univariate and multivariate analyses in this study demonstrated that the psychological help-seeking behaviours of migrant older adults were influenced by a combination of demographic, capability, opportunity, and motivational factors. Demographic characteristics, including gender and participation in reemployment or volunteer activities, significantly affected psychological help-seeking behaviours. Specifically, women were more likely to seek nonprofessional help than men were, which is consistent with the findings of Kong et al. (45), possibly due to lower social role expectations and reduced pressure related to emotional vulnerability. Compared with their nonengaged counterparts, older adults engaged in reemployment or volunteer work were more likely to seek help from nonprofessional sources; this may be explained by their broader social networks and greater access to informal support. These findings underscore the importance of encouraging migrant older adults to engage in reemployment or volunteer activities as a form of positive resocialization, which may indirectly promote psychological help-seeking behaviours.

In terms of capability-related factors, this study demonstrated that psychological distress and mental health literacy emerged as key predictors of psychological help-seeking behaviours. Distress has both positive and negative effects; whilst severe distress may motivate individuals to seek help due to somatic or emotional burdens—consistent with Kong et al. (45)—it may also lead to hopelessness and a reduced intention to seek help, as supported by Bretherton (49). Therefore, it is essential to improve awareness of mental health problems amongst older adults and their families, promote early symptom identification, and establish community-based follow-up mechanisms that offer timely interventions, including accessible online counselling services. Mental health literacy was also a strong predictor of professional psychological help-seeking behaviour, in line with Mackenzie and Pankratz (50). Individuals with higher literacy were more capable of recognising abnormal psychological symptoms, held stronger beliefs in the effectiveness of treatment, and were more adept at utilising available mental health resources. However, in this study, overall mental health literacy amongst migrant older adults was low and highly variable, particularly in the domains of treatment belief and awareness of resources, highlighting the need for accessible, culturally appropriate educational programmes and timely interventions, including online counselling services.

With respect to opportunity-related factors, this study showed that stigma towards professional psychological help seeking and the level of social support were found to be significantly associated with psychological help-seeking behaviour. In this study, older adults with lower levels of self-stigma were more inclined to seek professional help, echoing the findings of Mackenzie and Pankratz (50) and Murphy et al. (51). Whilst our data did not R1-5.

directly measure cultural beliefs, prior literature suggests that high levels of stigma in older Chinese adults may be rooted in Confucian values emphasising filial piety and hierarchical family structures, as well as beliefs such as “family matters should not be disclosed” (52). These cultural norms often discourage open discussion of emotional vulnerability, as older adults may perceive help-seeking as a sign of personal weakness or moral failing (53). This tendency is particularly pronounced in Huzhou, a city rich in cultural and historical heritage, where the accessibility of psychological assistance with privacy and dignity protection is also limited (54). Community efforts should focus on normalising mental health discussions and integrating psychoeducation into everyday settings to reduce shame and fear surrounding help seeking. In this study, social support was found to play a dual role in help-seeking behaviour, a finding that is consistent with previous studies by Bretherton (49) and Wang et al. (55). Whilst strong social networks facilitate nonprofessional psychological help seeking, they also buffer psychological distress, thereby reducing the perceived need for professional services. In this study, the level of social support was moderate to low, rooted in family caregiving roles and largely provided by family members and friends. Although such support facilitated nonprofessional help seeking and offered some emotional comfort, it was often passive and insufficient to address deeper psychological needs. In addition, structural barriers such as economic constraints, insurance barriers, limited community resource availability and corresponding social policy further hinder opportunities for professional help seeking, highlighting the need to improve affordability and continuity of services. Therefore, communities should develop diverse mental health support systems, including accessible peer networks and affordable services led by professionals or social workers, to provide safe, nonjudgemental spaces and reduce reliance on family support.

Motivational factors, particularly psychological help-seeking intention and attitudes towards professional psychological services, were found to be important predictors of both nonprofessional and professional psychological help-seeking behaviours in this study. Migrant older adults with stronger intentions were more likely to seek help, which is consistent with the findings of Huang et al. (40), indicating that intention is a key determinant of actual psychological help-seeking behaviour, especially for informal sources. Whilst intention is a crucial antecedent, it does not equate to actual behaviour, necessitating further exploration of the dynamic relationship between intention and action. In this study, the overall intention to seek help was low amongst participants, underscoring the urgent need to increase motivation through community-based mental health education, awareness campaigns, and peer support systems. Additionally, this study found that attitudes towards professional psychological help seeking significantly influenced behaviour. Many migrant older adults held pessimistic and sceptical views and emphasised self-reliance, which is consistent with the findings of Li (56). Such negative attitudes often reduced motivation to engage with professional support, limited willingness to explore available resources, and reinforced reliance on informal or nonprofessional sources. These suggest the need for public education, peer-led storytelling, and antistigma interventions to reshape perceptions and help migrant older adults recognise the legitimacy and necessity of seeking psychological help.

In summary, this study provides a comprehensive, theory-driven analysis of psychological help-seeking behaviour amongst migrant older adults, integrating demographic, capability, opportunity, and motivation factors within the COM-B framework, and highlighting both personal and structural determinants of help-seeking behaviour. The findings offer novel insights into help-seeking behaviour, mental health, and the experiences of migrant older adults, and provide empirical evidence to inform interventions and policy development targeting this vulnerable and rapidly growing population, whilst also extending the theoretical applicability and practical use of the COM-B framework.

## Limitations

The study has several limitations. First, regarding data representativeness, the data were collected exclusively from Huzhou City, Zhejiang Province, China, which may limit the generalizability of the findings; future studies should expand sample sizes and include multiregional comparisons. The convenience sampling method may have biassed the results towards older adults who are more socially active or more easily accessible, potentially limiting representativeness. The study sample had limited male representation, which may affect the interpretation of help-seeking patterns across genders. Second, regarding data collection and processing, the face-to-face, assistant-administered, or self-administered questionnaires may have introduced social desirability bias, measurement error, or minor data entry inaccuracies. Additionally, help-seeking scores, particularly for professional services, were highly skewed (median = 0), which may limit the sensitivity of the analyses and the interpretation of regression results. Third, regarding study design, whilst help-seeking intention was included as a predictor, it does not equate to actual behaviour, and the dynamic relationship between intention and action warrants further investigation. Finally, the cross-sectional design precludes causal inferences; thus, longitudinal studies are warranted to track dynamic changes in psychological help-seeking behaviour.

## Conclusion

Overall, migrant older adults exhibit a high prevalence of psychological distress but insufficient psychological help-seeking behaviours, particularly professional psychological help seeking. With respect to influencing factors, nonprofessional psychological help-seeking behaviour was associated mainly with demographic characteristics, capability, opportunity, and motivation, including gender, reemployment or volunteer participation, psychological distress, nonprofessional psychological help-seeking intention, mental health literacy, and social support. Professional psychological help-seeking behaviour is closely linked to psychological distress, professional psychological help-seeking intention, and mental health literacy. Future efforts should focus on enhancing mental health literacy and the accessibility of professional psychological services for migrant older adults; strengthening family and community support networks; and developing affordable, accessible, and acceptable psychological interventions to foster positive psychological help-seeking awareness and behaviours, thereby improving mental health status and overall well-being.

## Data Availability

The original contributions presented in the study are included in the article/supplementary material, further inquiries can be directed to the corresponding author.

## References

[1] Cao M Yao Q Liu H Mamman GII Wu T Zhao B . Development of a resocialization scale for Chinese older adult migrants. Res Gerontol Nurs. (2022) 15:245–53. doi: 10.3928/19404921-20220830-03, 36113011

[2] Haiyan L. New characteristic, trend and thinking of Chinese population——analysis based on data reported in seventh population census bulletin in 2020. J Qujing Normal Univ. (2021) 40:97. doi: 10.3969/j.issn.1009-8879.2021.04.019

[3] Zhou S Li K Ogihara A Wang X. Association between social capital and depression among older adults of different genders: evidence from Hangzhou, China. Front Public Health. (2022) 10:863574. doi: 10.3389/fpubh.2022.863574, 36033749 PMC9412187

[4] Lin X Haralambous B Pachana NA Bryant C LoGiudice D Goh A . Screening for depression and anxiety among older Chinese immigrants living in Western countries: the use of the geriatric depression scale (GDS) and the geriatric anxiety inventory (GAI). Asia Pac Psychiatry. (2016) 8:32–43. doi: 10.1111/appy.12191, 26010903

[5] Shi RB Hu HM. The impact of children structure on intergenerational social mobility expectations among pre elderly individuals. Popul J. (2024) 46:96–110. doi: 10.16405/j.cnki.1004-129X.2024.01.006

[6] Fang S Wang XQ Yang BX Liu XJ Morris DL Yu SH. Survey of Chinese persons managing depressive symptoms: help-seeking behaviours and their influencing factors. Compr Psychiatry. (2019) 95:152127. doi: 10.1016/j.comppsych.2019.152127, 31669791

[7] Ajzen I. The theory of planned behavior. Organ Behav Hum Decis Process. (1991) 50:179–211. doi: 10.1016/0749-5978(91)90020-T

[8] Rosenstock IM. Historical origins of the health belief model. Health Educ Monogr. (1974) 2:328–35. doi: 10.1177/109019817400200403 299611

[9] Goffman E. Stigma: Notes on the management of spoiled identity. New York: Simon and Schuster (2009).

[10] Putra BM Badayai ARA Soedjiwo NAF Suroyo S Guidi E Zakaria SM. Demographic factors as mediators between socio-psychological variables and psychological well-being in parents of children with down syndrome. Psikohumaniora: Jurnal Penelitian Psikologi. (2025) 10:139–58. doi: 10.21580/pjpp.v10i1.25583

[11] Barker G. Adolescents, social support and help-seeking behaviour. Geneva: World Health Organization; (2007) 10–11.

[12] Fu Y Lin W Yang Y Du R Gao D. Analysis of diverse factors influencing the health status as well as medical and health service utilization in the floating elderly of China. BMC Health Serv Res. (2021) 21:438. doi: 10.1186/s12913-021-06410-7, 33964906 PMC8106125

[13] Kim G Wang SY Park S Yun SW. Mental health of Asian American older adults: contemporary issues and future directions. Innov Aging. (2020) 4:igaa037. doi: 10.1093/geroni/igaa037, 33274302 PMC7691797

[14] Park NS Jang Y Chiriboga DA. Willingness to use mental health counseling and antidepressants in older Korean Americans: the role of beliefs and stigma about depression. Ethn Health. (2018) 23:97–110. doi: 10.1080/13557858.2016.1246429, 27764962

[15] Zhang X Yu B He T Wang P. Status and determinants of health services utilization among elderly migrants in China. Glob Health Res Policy. (2018) 3:8. doi: 10.1186/s41256-018-0064-0, 29568805 PMC5861660

[16] Jang Y Chiriboga DA Park NS Yoon H Cho YJ Hong S . The role of self-rated mental health in seeking professional mental health services among older Korean immigrants. Aging Ment Health. (2021) 25:1332–7. doi: 10.1080/13607863.2020.1758908, 32349527 PMC7606462

[17] Wu HL Xue YZ Wu JY. A visualized analysis of research on migrant older adults from 2010 to 2020. Nurs Res. (2022) 36:2078–85. (In Chinese). doi: 10.12102/j.issn.1009-6493.2022.12.003

[18] Chen S Shang S Qi L Liu T Yin J Song L . Research hotspots and trends in the field of mental health of migrant elderly at home and abroad based on CiteSpace. Chin Gen Pract. (2024) 27:3803. doi: 10.12114/j.issn.1007-9572.2023.0496

[19] Liu S Qin B Wang D. How does social integration work when older migrants obtain health services from community? Evidence from national database in China. Front Public Health. (2023) 11:1283891. doi: 10.3389/fpubh.2023.1283891, 38192547 PMC10773583

[20] Fonseca A Gorayeb R Canavarro MC. Women′s help-seeking behaviours for depressive symptoms during the perinatal period: socio-demographic and clinical correlates and perceived barriers to seeking professional help. Midwifery. (2015) 31:1177–85. doi: 10.1016/j.midw.2015.09.002, 26433622

[21] Koutra K Pantelaiou V Mavroeides G. Breaking barriers: Unraveling the connection between mental health literacy, attitudes towards mental illness, and self-stigma of psychological help-seeking in university students. Psychol Int. (2022) 6:590–602. doi: 10.3390/psycholint6020035, 41291439

[22] Elshaikh U Sheik R Saeed RKM Chivese T Alsayed Hassan D. Barriers and facilitators of older adults for professional mental health help-seeking: a systematic review. BMC Geriatr. (2023) 23:516. doi: 10.1186/s12877-023-04229-x, 37626290 PMC10463345

[23] Found A. Relationship between traditional Chinese beliefs about aetiology of mental disorders and help seeking: a survey of the elderly in Macao. East Asian Arch Psychiatr. (2016) 26:3–9. 27086754

[24] Chai YH Tong SF Khairuddin W. Intention to seek professional help for depression and its associated factors among elderly patients in Tenkera health clinic, Melaka, Malaysia. Med J Malaysia. (2021) 76:61–7. 33510111

[25] Sorkin DH Murphy M Nguyen H Biegler KA. Barriers to mental health Care for an Ethnically and Racially Diverse Sample of older adults. J Am Geriatr Soc. (2016) 64:2138–43. doi: 10.1111/jgs.14420, 27565017 PMC5937991

[26] Khatib HE Alyafei A Shaikh M. Understanding experiences of mental health help-seeking in Arab populations around the world: a systematic review and narrative synthesis. BMC Psychiatry. (2023) 23:324. doi: 10.1186/s12888-023-04827-4, 37161342 PMC10170733

[27] Mojaverian T Hashimoto T Kim HS. Cultural differences in professional help seeking: a comparison of Japan and the u.s. Front Psychol. (2013) 3:615. doi: 10.3389/fpsyg.2012.00615, 23426857 PMC3576055

[28] Zhang J. A study on the relationship among stigma of mental illness, perceived social support, and attitudes toward seeking professional psychological help in college students. Kaifeng: Henan University (2020).

[29] Putra BM Badayai ARA. Psychological well-being of parents with down syndrome children: the role of demographics. e-Bangi J Soc Sci Human. (2025) 22:550–60. doi: 10.1016/j.cpr.2024.102426

[30] Michie S Van Stralen MM West R. The behaviour change wheel: a new method for characterising and designing behaviour change interventions. Implement Sci. (2011) 6:42. doi: 10.1186/1748-5908-6-42, 21513547 PMC3096582

[31] Teo K Churchill R Riadi I Kervin L Cosco T. Help-seeking behaviours among older adults: a scoping review protocol. BMJ Open. (2021) 11:e043554. doi: 10.1136/bmjopen-2020-043554, 33593783 PMC7888354

[32] Liu J Zhang Y. Understanding and facilitating mental health help-seeking of young adults: a socio-technical ecosystem framework. arXiv. (2024). Available online at: https://arxiv.org/abs/2401.08994

[33] Ma R Wang Y Wang XQ Yu K Zhang CC Zhou YQ. Analysis of hindering and facilitating factors of help-seeking behavior in schizophrenia based on COM-B model: a descriptive qualitative study. BMC Psychiatry. (2023) 23:770. doi: 10.1186/s12888-023-05226-5, 37867190 PMC10591348

[34] Nguyen S Blake J Ng F Patterson S. 'Who you gonna call?' a qualitative study of psychiatrists accessing mental health services. Australas Psychiatry. (2024) 32:157–63. doi: 10.1177/10398562231222767, 38127794

[35] Cheng M Duan C. The changing trends of internal migration and urbanization in China: new evidence from the seventh National Population Census. China Popul Dev Stud. (2021) 5:275–95. doi: 10.1007/s42379-021-00093-7

[36] Cao M Wang Y Liu H Li Q. Factors influencing the resocialization of migrant older adults in China: a cross-sectional study. Front Public Health. (2025) 13:1511838. doi: 10.3389/fpubh.2025.1511838, 40302777 PMC12037397

[37] Ni P Chen L Liu N. The sample size estimation in quantitative nursing research. Chin J Nurs. (2010) 45:378–80.

[38] Gong X Xie XY Xu R Luo YJ. Evaluation report of the Chinese version of the depression anxiety stress scales (DASS-21) among Chinese college students. Chin J Clin Psychol. (2010) 18:443–6. (In Chinese). doi: 10.16128/j.cnki.1005-3611.2010.04.020

[39] Wilson CJ Deane FP Ciarrochi J Rickwood D. Measuring help-seeking intentions: properties of the general help seeking questionnaire. Can J Couns. (2005) 39:15–28.

[40] Huang S. Study on psychological help-seeking behavior and its influencing factors among perinatal women. Wuhan: Huazhong University of Science and Technology (2022).

[41] Fang S Yang BX Yang F Zhou Y. Reliability and validity of the Chinese version of the attitudes toward seeking professional psychological help scale – short form among community residents. Nurs Res. (2019) 33:2410–4. (In Chinese). doi: 10.12102/j.issn.1009-6493.2019.14.009

[42] Hao ZH Liang BY. Revision of the stigma scale for seeking professional psychological help in college students. Chin Ment Health J. (2011) 25:646–9. (In Chinese). doi: 10.3969/j.issn.1000-6729.2011.09.002

[43] Ming ZJ Chen ZY Wang YX Jiang L Guo F. Reliability and validity evaluation of the Chinese version of the multicomponent mental health literacy measure among male military personnel. Chin J Public Health. (2021) 37:86–91 (In Chinese). doi: 10.11847/zgggws1123418

[44] Huang L Jiang QJ Ren WH. Correlation among coping style, social support, and psychosomatic symptoms in cancer patients. Chin Ment Health J. (1996) 10:160–6.

[45] Kong D Guo M Simon M Dong X. Immigration-related factors and depression help-seeking behaviors among older Chinese Americans. Clin Gerontol. (2024) 48:270–9. doi: 10.1080/07317115.2024.2348051, 38695308

[46] Liang YJ Deng F Liang P Zhong BL. Suicidal ideation and mental health help-seeking behaviors among older Chinese adults during the COVID-19 pandemic. J Geriatr Psychiatry Neurol. (2022) 35:245–51. doi: 10.1177/08919887221078568, 35139677 PMC8844439

[47] Pérez-Zepeda MU Arango-Lopera VE Wagner FA Gallo JJ Sánchez-García S Juárez-Cedillo T . Factors associated with help-seeking behaviors in Mexican older individuals with depressive symptoms: a cross-sectional study. Int J Geriatr Psychiatry. (2013) 28:1260–9. doi: 10.1002/gps.3953, 23585359 PMC3797168

[48] Kim W Kreps GL Shin CN. The role of social support and social networks in health information-seeking behavior among Korean Americans: a qualitative study. Int J Equity Health. (2015) 14:40. doi: 10.1186/s12939-015-0169-8, 25927546 PMC4419489

[49] Bretherton SJ. The influence of social support, help seeking attitudes and help seeking intentions on older Australians’ use of mental health services for depression and anxiety symptoms. Int J Aging Hum Dev. (2022) 95:308–25. doi: 10.1177/00914150211050882, 34747228

[50] Mackenzie CS Pankratz L. Perceived need, mental health literacy, neuroticism and self-stigma predict mental health service use among older adults. Clin Gerontol. (2025) 48:161–74. doi: 10.1080/07317115.2022.2058440, 35400301

[51] Murphy DJ Mackenzie CS Dryden RP Hamm JM. Perceived control moderates the internalized stigma model of seeking mental health services in distressed older adults. J Couns Psychol. (2024) 71:473–86. doi: 10.1037/cou0000733, 38602789 PMC11954132

[52] Yang H Hagedorn A Zhu H Chen H. Mental health and well-being in older women in China: implications from the Andersen model. BMC Geriatr. (2020) 20:254. doi: 10.1186/s12877-020-01639-z, 32711464 PMC7382081

[53] Dong X Zhang M. The association between filial piety and perceived stress among Chinese older adults in greater Chicago area. J Geriatr Palliat Care. (2016) 4:10.13188/2373-1133.1000015. doi: 10.13188/2373-1133.1000015, 27642631 PMC5026127

[54] Yang J Li Y Gao R Chen H Yang Z. Relationship between mental health literacy and professional psychological help-seeking attitudes in China: a chain mediation model. BMC Psychiatry. (2023) 23:956. doi: 10.1186/s12888-023-05458-5, 38129805 PMC10734200

[55] Wang X Bergren S Dong X. Association between social support and depression help seeking behaviors among US Chinese older adults. J Aging Health. (2023) 35:83–93. doi: 10.1177/08982643221106870, 35694797

[56] Li FL. The public perception of mental illness in China: Content, structure, and measurement. Wuhan: Central China Normal University (2015).

[57] Lovibond PF Lovibond SH. The structure of negative emotional states: comparison of the Depression Anxiety Stress Scales (DASS) with the beck depression and anxiety inventories. Behav Res Ther. (1995) 33:335–43. doi: 10.1016/0005-7967(94)00075-u 7726811

